# Reliability of Muscle Oxygen Saturation for Evaluating Exercise Intensity and Knee Joint Load Indicators

**DOI:** 10.3390/jfmk10020136

**Published:** 2025-04-17

**Authors:** Aldo A. Vasquez-Bonilla, Rodrigo Yáñez-Sepúlveda, Matías Monsalves-Álvarez, Marcelo Tuesta, Daniel Duclos-Bastías, Guillermo Cortés-Roco, Jorge Olivares-Arancibia, Eduardo Guzmán-Muñoz, José Francisco López-Gil

**Affiliations:** 1 Faculty of Sport Sciences, University of Extremadura, 10001 Caceres, Spain; alvasquezb@unex.es; 2 Faculty Education and Social Sciences, Universidad Andres Bello, Viña del Mar 2520000, Chile; rodrigo.yanez.s@unab.cl; 3 Exercise and Rehabilitation Sciences Institute, Faculty of Rehabilitation Sciences, Universidad Andres Bello, Santiago 7550000, Chile; matias.monsalves@unab.cl (M.M.-Á.); marcelo.tuesta@unab.cl (M.T.); 4 Geroscience Center for Brain Health and Metabolism (GERO), Santiago 8320000, Chile; 5 Laboratory of Sport Sciences, Sports Medicine Centre “Sports MD”, Viña del Mar 2520000, Chile; 6 iGEO Group, School of Physical Education, Pontificia Universidad Católica de Valparaíso, Valparaíso 2340000, Chile; daniel.duclos@pucv.cl; 7 IGOID Research Group, Faculty of Sport Science, University of Castilla-La Mancha, 45071 Toledo, Spain; 8 Faculty of Life Sciences, Universidad Viña del Mar, Viña del Mar 2520000, Chile; guillermo.cortes@uvm.cl; 9 Grupo AFySE, Investigación en Actividad Física y Salud Escolar, Escuela de Pedagogía en Educación Física, Facultad de Educación, Universidad de las Américas, Santiago 8320000, Chile; jolivares@udla.cl; 10 Escuela de Kinesiología, Facultad de Salud, Universidad Santo Tomás, Talca 3460000, Chile; eguzmanm@santotomas.cl; 11 Escuela de Kinesiología, Facultad de Ciencias de la Salud, Universidad Autónoma de Chile, Talca 3460000, Chile; 12 One Health Research Group, Universidad de Las Américas, Quito 170124, Ecuador

**Keywords:** skeletal muscle, knee, exercise, oxygen uptake, angular velocity

## Abstract

**Objectives:** This study aimed to evaluate the reliability of muscle oxygen saturation (SmO_2_) and its correlation with variables from an inertial measurement unit (IMU) sensor placed on the knee at different exercise intensities. **Methods:** Fourteen university athletes participated in the study. Incremental ergospirometry was performed to exhaustion to calculate V’O_2_max, determine training zones, heart rate, and workload using the IMU, and analyze muscle metabolism by SmO_2_. **Results:** The analysis revealed significant differences between moderate-to-high-intensity zones (80–89% vs. 50–69%, Δ = 27% of SmO_2_; *p* < 0.001) and high-intensity zones (90–100% vs. 50–79%, Δ = 35% of SmO_2_; *p* < 0.001). SmO_2_ values showed moderate reliability at moderate exercise intensities (e.g., ICC 0.744 at 50%) and high variability at higher intensities, with ICC values around 0.577–0.594, and CV% increasing up to 77.7% at 100% intensity, indicating decreasing consistency as exercise intensity increases. SmO_2_ significantly decreases with increasing angular velocity (β = −13.9, *p* < 0.001), while knee joint load only shows significant correlations with SmO_2_ in the moderate-to-high-intensity zones (r = 0.569, *p* = 0.004) and high-intensity zones (r = 0.455, *p* = 0.012). **Conclusions:** SmO_2_ is a key predictor of performance during maximal incremental exercise, particularly in high-intensity zones. Moreover, SmO_2_ has the potential to serve as a physiological marker of the internal load on the muscles surrounding the knee during exercise. The SmO_2_ decrease could depend on the angular velocity and impact of the exposed knee during running.

## 1. Introduction

Non-invasive near-infrared spectroscopy (NIRS) sensors have been used to measure muscle oxygen saturation (SmO_2_) in sports science and are gaining popularity in performance and health research [1]. SmO_2_ is defined as the balance between oxygen delivery and extraction by muscles and can serve as an indicator of exercise intensity [2]. It also helps identify peripheral fatigue tolerance, as reduced muscle oxygenation is associated with decreased contractile capacity and increased metabolite accumulation [3]. Moreover, using SmO_2_ as a metabolic marker of internal load when combined with external load measures, such as speed and endurance, can provide valuable insights into overall training load [4].

Numerous studies have validated the use of SmO_2_ as an intensity parameter [1], emphasizing its application in endurance tests, such as cycling and running [5,6]. Research has demonstrated that SmO_2_ using NIRS sensors provide reliable measurements during low-to-moderate-intensity exercises [6]. They can be used in conjunction with V’O_2_ to enhance our understanding of how the amount of oxygen consumed by the body correlates with that absorbed by the muscles [7]. Additionally, SmO_2_ has been studied across various exercise intensity levels, revealing strong correlations between SmO_2_ and lactate equivalence in the gastrocnemius and vastus lateralis muscles [8]. Furthermore, during high-intensity efforts, data analysis tends to exhibit non-linear behavior, allowing SmO_2_ to identify critical power or repeated sprint ability [9,10].

Muscle strength is a key indicator for optimizing both performance and health [11]. Research has shown that muscle strength is inversely correlated with SmO_2_ in the vastus lateralis during high-intensity contractions, suggesting that an SmO_2_ decrease may be a sign of improved energy transport and fatigue tolerance [3]. Furthermore, it is theorized that SmO_2_ measured in the knee extensor and flexor muscles could be useful for assessing injury risk in athletes [12]. Moreover, SmO_2_ in the vastus lateralis has been associated with horizontal strength in athletes [10]. In this regard, a key component of reconditioning involves monitoring external load using inertial measurement unit (IMU) sensors to track the player’s speed, acceleration, and load during physical rehabilitation [13,14]. IMUs can detect angular velocities, force, and acceleration during walking and running, making them helpful in assessing joint support capacity during injury rehabilitation (e.g., in the knee) [15]. Variables such as angular velocity and different force vectors are used to complement the biomechanical analysis of changes in knee position during exercise [16]. Another strength of IMU sensors is their low cost and wide applicability in exercise science [17]. However, it remains unclear whether SmO_2_ levels in the vastus lateralis at different intensities correlates with external load on the knee during running.

In this context, it remains unclear whether improvements in oxygen extraction by the muscle are directly related to the IMU parameters in the knee joint [18]. Additionally, the relationship between SmO_2_ and surface electromyography (EMG) shows a linear correlation with the flexion moment [19]. Also, the changes in spiroergometric parameters (ventilatory thresholds) have been shown to influence muscle oxygenation levels, affecting muscle recruitment responses [20]. Despite these insights, studying the reliability and relationship between SmO_2_ and IMU sensors is valuable due to the ease of transporting these sensors and obtaining daily data during training. This practicality makes them a highly useful tool for coaches in their everyday practice.

Therefore, the possible relationship of SmO_2_ with IMU variables (acceleration, angular velocity, and knee joint load) and exercise intensity exposed by spiroergometric parameters could improve the understanding of SmO_2_ analysis for future applications in rehabilitation and sports performance. This study aimed to evaluate the reliability and correlation of SmO_2_ with IMU sensor variables placed on the knee at different intensities. It was hypothesized that increasing intensity would increase knee joint load and might be associated with decreased SmO_2_ in athletes.

## 2. Materials and Methods

### 2.1. Problem Experimental

This study was cross-sectional with a test–retest and correlational design. The objective was to determine the reliability of SmO_2_ measurements at different intensities during an exercise incremental treadmill test. Additionally, SmO_2_ was correlated with external load (IMU) parameters. In this regard, the use of SmO_2_ as a marker of internal load remains debated, as more practical data exploration is needed. It is also unclear whether SmO_2_ depends on variables such as force, acceleration, and angular velocity at different intensities during a treadmill test commonly used to determine training zones in athletes. This study provided valuable insights into energy metabolism, helping to personalize training loads and optimize performance and health using NIRS sensors.

### 2.2. Participants

Fourteen physically active men were recruited for this study (mean age: 22.7 ± 3.5 years; height: 1.72 ± 9.01 m; weight: 69.8 ± 11.3 kg; BMI: 24.1 ± 2.4 kg/m^2^; body fat: 13.6 ± 4.3%). Participants were university athletes with 1 to 3 years of experience, comprising 4 handball players, 4 sprinters (100–400 m), and 6 futsal players.

The inclusion criteria required that they engaged in training activities more than three days a week and did not have any metabolic diseases that could affect the test results. Additionally, participants who reported any type of lower limb injury in the month prior to the study were excluded. Each participant provided written informed consent based on the approval of the protocol by the ethics committee of the University of Viña del Mar (registration number: Code R62-19a, approved on 27 January 2020).

### 2.3. Protocol

**Figure 1:**
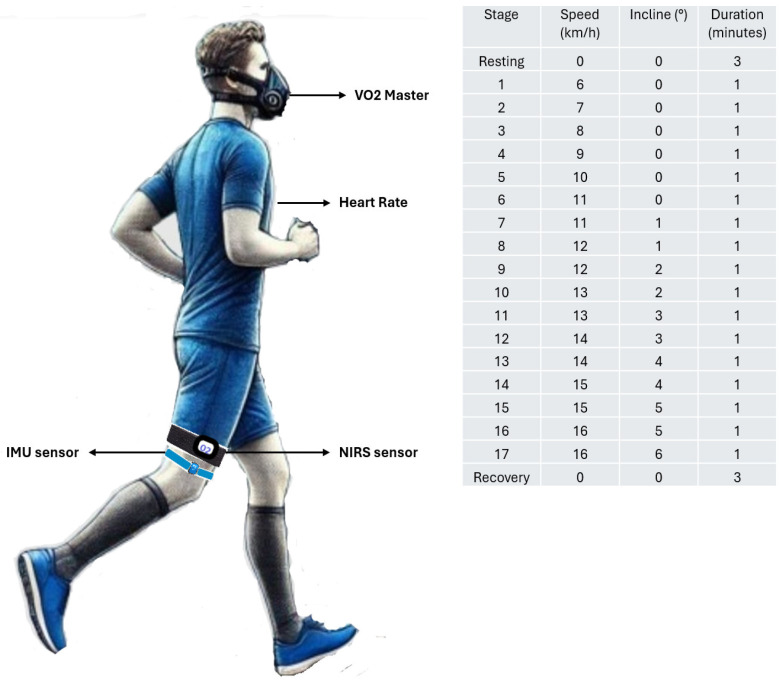
Description of the location of V’O_2_ Master, IMU, and NIRS sensors during the incremental exercise protocol.

Before the incremental test, participants rested for 3 min to calibrate all sensors and the V’O_2_ Master device to baseline conditions. The test began at a speed of 6 km/h, with increments of 1 km/h each minute until reaching 11 km/h. Beyond this point, the treadmill incline was increased by 1 degree per stage, with each stage lasting 2 min; this represents a 2% increase every 2 min until the subjects either reached volitional exhaustion [21,22] or attained a maximum speed of 16 km/h at a 6-degree incline, which marked the end of the test. Figure 1 illustrates the protocol design.

Furthermore, to confirm that the test met the maximal exhaustion criteria, the V’O_2_ max criterion described by Lacour et al. [23] was applied. This criterion is defined as the highest plateau achieved, identified by two consecutive maxima within 150 mL·min^−1^, with data averaged over 5-s intervals. Additionally, participants reported a subjective effort level between 18 and 20 on the Borg scale (6–20) [21,22]. Finally, a passive recovery period of 3 min was implemented at the end of the test [24,25].

All tests were performed in a physiology laboratory specializing in ergospirometry. The room was maintained at a temperature of approximately 22–24 °C with a relative humidity of 40–50%. Athletes were scheduled for testing between 8:00 AM and 2:00 PM and were divided into two test groups (Monday and Tuesday) to ensure sufficient measurement time for each participant. Each participant was tested twice (retest), with the second test conducted one week after the first test and at the same time of day to maintain consistent circadian conditions. To minimize potential bias, the following criteria were established: (a) a minimum of 48 h of rest or abstention from high-intensity exercise, and (b) participants were instructed to maintain their usual sleep habits to avoid any decrease in performance.

### 2.4. Assessment

#### 2.4.1. V’O_2_ Master Pro Analyzer

The V’O_2_ Master Pro (version 1.1.1) was used for the test protocol. The device, weighing 0.32 kg, was powered by a single AAA battery. It was connected to a Hans Rudolph 7450 V2 mask via a manufacturer-supplied “wearpiece” adapter, which includes an exhaust port for air flow (30–160 L/min) and a single-use filter that was replaced after each trial. A soft harness secured the unit to the participants’ faces. The total dead space of the mask–wearpiece system was ~125 mL [26].

The V’O_2_ Master was automatically calibrated to ambient air for gas concentrations, temperature, humidity, and barometric pressure upon activation but was not calibrated to other O_2_ concentrations. After calibration, participants took 10–15 deep breaths to calibrate the flowmeter. The device measured breath-by-breath ventilation, from which minute ventilation (V’E) and V’O_2_ were derived, but it did not measure V’CO_2_ due to the absence of a CO_2_ sensor. Data were transmitted via Bluetooth to an iPad (version 11, Apple, Cupertino, CA, USA) equipped with the V’O_2_ Master mobile app for storage and subsequent download. Participants also wore a Polar H10 heart rate monitor (Polar Electro Oy, Kempele, Finland) placed below the xiphoid process. Real-time heart rate data were transmitted to the V’O_2_ Master app and later analyzed in Excel (Excel 2024, Microsoft Office 365, Microsoft, Redmond, WA, USA).

#### 2.4.2. Exercise Intensity

Aerobic intensity was defined in the following percentages: 50–59% (walking), 60–69% (warm-up/cooldown, easy long runs), 70–79% (long runs, uphill runs, progressive runs), 80–89% (threshold runs/intervals, fartlek, competitions), 90–99% (V’O_2_max intervals, competitions, hill repetitions), and 100% as the maximum value reached during the test, sustained for 30 s at the end of the trial [27,28]. The average data from the last 30 s of each intensity stage in the protocol were used.

To calculate the percentage of V’O_2_max (%$\dot{V}$O_2_max) based on the maximum V’O_2_ reached during the test, the following formula was used: (Percentage of V’O_2_ ÷ 100) × V’O_2_max [27]. Additionally, for metabolic zone analysis, SmO_2_ data were evaluated according to the following zones: LIT = low-intensity training (50–79%), MIT = moderate-intensity training (80–90%), and HIT: high-intensity training (>90%) [28].

#### 2.4.3. Inertial Measurement Unit (IMU)

To evaluate the external load, a wireless inertial sensor (WT9011DCL, WitMotion, Shenzhen, China) was used. This sensor integrates a three-axis gyroscope, a three-axis accelerometer, and a three-axis magnetometer. The device has dimensions of 51.3 mm × 36 mm × 15 mm and a net weight of 9 g. The Witmotion app was employed to connect the IMU to an iPad (version 11, Apple), with a wireless coverage range of 50 m under optimal conditions (i.e., no obstacles). The app enables the retrieval and storage of data in CSV format, including measurements from accelerometers, gyroscopes, and magnetometers. The recorded variables included acceleration (m/s^2^), angular velocity (°/s), and knee joint load, represented by the total sum of accelerations in their vectors (see Formula (1)), expressed in arbitrary units. Knee joint load was calculated using the following formula [29]:

(1) $$Load=\frac{\sqrt{{\left(ay(t)-ay(t-1\right)}^{2}+{\left(ax(t)-ax(t-1\right)}^{2}+{\left(az(t)-az(t-1\right)}^{2}}}{100}$$

The sensor was placed laterally near the knee, approximately 3 cm above the knee joint axis [30]. Fixation was achieved using a tight-fitting garment designed for physical activity, supplemented with kinesiology tape to enhance stability and reduce motion artifacts during movement. The knee joint angle was modeled as a 3D rigid body, approximating the motion of the human leg. Raw data from the IMU were collected at a sampling rate of 10 Hz [31,32,33].

#### 2.4.4. Muscle Oxygen Saturation Through of Near-Infrared Spectroscopy (NIRS) Technology

Local SmO_2_ assessment was carried out using a NIRS sensor (Moxy, Fortiori Design LLC, Minneapolis, MN, USA) with a sampling rate of 1 Hz, which is reliability for measuring SmO_2_ (ICC = 0.773–0.992) [6]. It was firmly attached to the belly of the right vastus lateralis muscle (midway between the lateral epicondyle and the greater trochanter of the femur) using a dark elastic strap to avoid light contamination and motion artifacts. Skinfold thickness at the NIRS measurement site (vastus lateralis) was measured using a skinfold caliper (Harpenden Lange Skinfold Caliper, Cambridge Scientific Industries, Inc., Cambridge, MD, USA) to ensure that the skinfold thickness was <1/2 the distance between the emitter and the detector (25 mm) [34].

The following guidelines were adhered to for data analysis: (1) average values for each minute of data collection were used; (2) any SmO_2_ values exceeding 10% after the last recorded value were excluded; and (3) readings showing 0% were also excluded due to apparent signal loss. Real-time data were visible to the NIRS technology research expert via Bluetooth and transferred to a Garmin system (Forerunner 735xt, Garmin, Olathe, KS, USA). Each test was analyzed using Excel (Excel 2024, Microsoft Office 365, Microsoft).

### 2.5. Statistical Analysis

The variables are described as mean ± standard deviation (SD). The Shapiro–Wilk normality test was applied to each variable. When normality was confirmed, a one-way ANOVA was conducted to compare the different exercise intensities, followed by a Tukey post hoc test to identify significant differences between steps and metabolic zones (LIT, MIT, and HIT). The reliability of measurements between trials was assessed using the coefficient of variation (CV), calculated as CV = SD ÷ mean × 100, and the intraclass correlation coefficient (ICC) (two-way random effects model for absolute agreement). ICC values were interpreted as follows: poor (≤0.50), moderate (0.50–0.75), good (0.76–0.90), and excellent (>0.90) reliability [35]. Additionally, the standard error of measurement (SEM) was calculated as SEM = SDdiff ÷ √2, where SDdiff is the standard deviation of the differences between tests. The minimum detectable change (MDC) was calculated from the SEM at a 95% confidence interval (CI) using the formula MDC = SEM × 1.96 × √2, based on the approach of Hopkins [36]. The SEM was compared to the MDC as proposed by Liow and Hopkins [37], with the following criteria: good sensitivity for SEM < MDC, satisfactory for SEM = MDC, and marginal for SEM > MDC. A Pearson correlation test was used to evaluate the relationship between spiroergometric and IMU parameters with SmO_2_. The magnitude of the Pearson correlation was interpreted as suggested by Hopkins et al. [38]: 0.0–0.1 (trivial), 0.1–0.3 (small), 0.3–0.5 (moderate), 0.5–0.7 (large), 0.7–0.9 (very large), and 0.9–1.0 (almost perfect). The power of each variable was calculated using G * Power statistical software (Düsseldorf, Germany v3.1.3, 3). The interpretation of g power was calculated between 0.8 and 1, indicating sufficient statistical power. Linear regression analyses were performed to further assess relationships between variables. The first regression model examined the relationship between spiroergometric parameters, IMU variables, and SmO_2_, as independent variables, and test time, as the dependent variable. A second regression model evaluated the relationship between IMU parameters, as independent variables, and SmO_2_, as the dependent variable. Assumptions of the regression models—normality, homoscedasticity, and the absence of multicollinearity—were verified to ensure the validity of the analysis. Results were reported using standardized beta coefficients and *p*-values, with a significance threshold set at *p* < 0.05. Significance levels for Pearson correlations and linear regression analyses were calculated based on the full dataset, comprising multiple repeated measures per subject (i.e., 12 data points per subject across exercise intensity zones, with two assessments per subject, totaling 168 data points from 14 subjects). All data analyses were performed using JAMOVI version 2.2 (The Jamovi Project, 2020) [39].

## 3. Results

Table 1 presents the mean values and standard deviations of the physiological and IMU variables during the maximal incremental test. Initially, differences were observed only in the physiological parameters comparing resting values to the start of the test in V’O_2_ (mL/min), V’O_2_ (mL/kg/min), V’E (L/min), HR (bpm) (*p*-value < 0.001), and SmO_2_ (*p*-value = 0.004). The external load IMU variables (acceleration, knee joint load, and angular velocity) showed no differences compared to the previous stage. According to the analysis of SmO_2_ across the training zones, a significant difference was observed between MIT and LIT (80–89% vs. 50–69%, 27% difference; *p* < 0.001) and between HIT and LIT (90–100% vs. 50–79%, 35% difference; *p* < 0.001). However, no differences were observed between HIT and MIT zones.

Furthermore, the results showed sufficient statistical power in V’O_2_ (g power = 0.91), V’O_2_max (g power = 0.92), V’E (g power = 0.88), and HR (g power = 0.92), but no high statistical power was seen in SmO_2_ (g power = 0.64), acceleration (g power = 0.72), knee joint load (g power = 0.70), and angular velocity (g power = 0.74).

Table 2 presents the reliability and sensitivity analysis of SmO_2_ values across different exercise intensities. At rest, SmO_2_ values had a mean of 57.9 ± 15.1 in the first test and 64.0 ± 11.5 in the second, with a low ICC (0.143) and a CV% of 21.8. Sensitivity analysis showed an SE of 3.5% and an MDC of 9.7%. SmO_2_ values progressively decreased as exercise intensity increased, with moderate reliability observed at moderate intensities. For instance, at 50% intensity, the ICC was 0.744 with a CV% of 23.7, while at 60%, the ICC dropped to 0.527, and the CV% increased to 29.2. At high intensities, such as 80% and 90%, similar ICC values were observed (0.577 and 0.594, respectively), but CV% values were considerably higher (33.6 and 50.2, respectively), indicating greater variability in these zones. Sensitivity in these zones showed lower SE values, such as 1.6 at 80% and 1.2 at 90%, with MDC values of 4.4 and 3.3, respectively. Finally, at 100%, SmO_2_ values were 9.1 ± 7.4 in the first test and 10.2 ± 7.6 in the second, with an ICC of 0.729 but a high CV% (77.7), suggesting lower consistency in this maximal intensity zone.

Table 3 presents a correlation analysis between spiroergometric parameters, IMU variables, and SmO_2_. The results show statistically significant correlations (*p* < 0.05) between SmO_2_ and the other variables analyzed, with consistent negative relationships observed with both spiroergometric parameters and IMU variables. Specifically, SmO_2_ exhibited strong inverse relationships with VO_2_ (r = −0.799), VE (r = −0.800), and HR (r = −0.783). Regarding the IMU sensor variables, SmO_2_ demonstrated moderate negative correlations with acceleration (r = −0.455) and knee joint load (r = −0.379). Additionally, a stronger negative correlation was observed with angular velocity (r = −0.617), highlighting a relationship between movement patterns and local muscular oxygen consumption.

The Table 4 shows the linear regression analysis, with time as the dependent variable, indicates that several physiological predictors have a significant effect (*p* < 0.05), and the model explains 73% of the variance in test performance (R^2^ = 0.734, r = 0.862). Among the predictors, VO_2_ showed the highest positive contribution to performance (β = 10.2, *p* < 0.001). Conversely, SmO_2_ (β = −3.7, *p* < 0.001) exhibited negative associations, suggesting that greater SmO_2_ utilization is indicative of better performance during incremental running. In contrast, VE, HR, acceleration, knee joint load, and angular velocity were not significant predictors.

**Figure 2:**
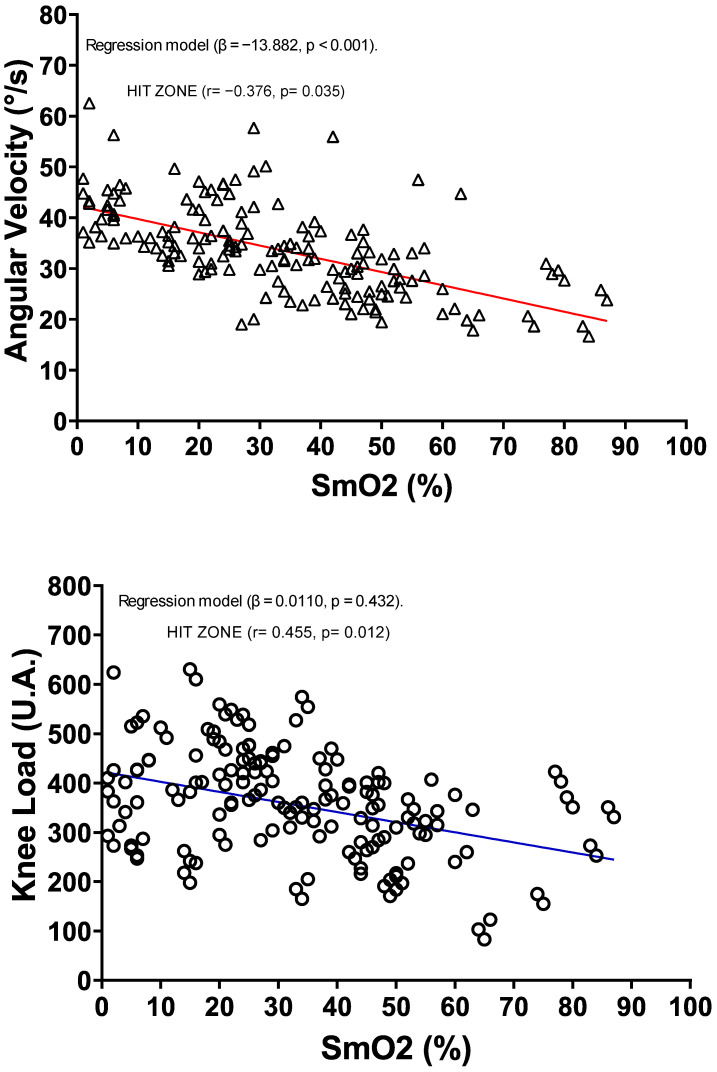
Association between muscle oxygen saturation and angular velocity during a maximal incremental exercise test. The linear regression analysis between the variables of the angular velocity and SmO_2_ represented by triangles, and knee load and SmO_2_ represented by circles.

Figure 2 illustrates the relationship between SmO_2_ with knee joint load and angular velocity. The linear regression analysis reveals that, among the external load variables measured by the IMU, only angular velocity showed a significant association (β = −13.9, *p* < 0.001). This indicates that an increase in angular velocity is associated with a SmO_2_ decrease. In contrast, knee joint load did not significantly predict a decrease in SmO_2_ (β = 0.011, *p* = 0.432).

Additionally, correlation analysis results indicated that SmO_2_ did not have a significant relationship with angular velocity (r = 0.322, *p* = 0.063) or knee joint load (r = −0.055, *p* = 0.399) in the LIT zone. In the MIT zone, angular velocity also did not exhibit a significant relationship (r = 0.173, *p* = 0.209), whereas knee joint load showed a significant correlation (r = 0.569, *p* = 0.004). Finally, in the HIT zone, SmO_2_ showed significant correlations with both angular velocity (r = −0.376, *p* = 0.035) and knee joint load (r = 0.455, *p* = 0.012).

## 4. Discussion

The main finding of the study is that SmO_2_ is a significant physiological marker for identifying performance in a maximal incremental test. Additionally, there is a moderate inverse relationship between angular velocity and SmO_2_ levels during high-intensity exercise. These results may inform future studies evaluating the interaction between SmO_2_ in the muscles surrounding the knee joint load.

The analysis of SmO_2_ across different training zones (see Table 1) revealed significant differences between MIT and LIT zones (80–89% vs. 50–69%, Δ = 27% of SmO_2_; *p* < 0.001). There were also notable differences between HIT and LIT zones (90–100% vs. 50–79%, Δ = 35% of SmO_2_; *p* < 0.001). These findings are consistent with previous research, which indicates that SmO_2_ levels decrease progressively as exercise intensity increases due to greater oxygen extraction by the working muscles [6,40]. However, the absence of significant differences between HIT and MIT suggests a plateau effect in SmO_2_ at higher intensities [9]. This marked SmO_2_ decrease is crucial for supporting high-intensity running and is associated with improved performance [1].

Also, the results demonstrate that the reliability of SmO_2_ depends on exercise intensity (see Table 2). At rest, the values show low consistency (ICC = 0.143, CV = 21.8%), but at 50% intensity, reliability improves significantly (ICC = 0.744, CV = 27%). However, as intensity increases, reliability declines (ICC = 0.527 to 0.594), with higher data variability at higher intensities. These findings are consistent with those from Crum et al. [6], who reported better reliability at lower intensities compared to high intensities (r = 0.773–0.992), and Yogev et al. [41], who reported ICC values for SmO_2_ of 0.81–0.90 across exercise intensities. These studies are comparable to ours, since they used the same MOXY monitor sensor on the vastus lateralis muscle. However, the protocols in those studies were performed on a bicycle, which may yield more consistent results compared to using a treadmill [40]. Running shows greater SmO_2_ data variability, mainly when measured in the rectus femoris [18] and gastrocnemius muscles [5]. Although low reliability suggests a limitation in the precision of NIRS sensors as intensity markers, sensitivity (SEM and MDC) indicates that the biological noise errors of NIRS in identifying training adaptations may range between 6 and 10% and decrease as intensity increases [42]. This suggests that SmO_2_ changes >11% should be considered to account for variability caused by fatigue [40]. Along the same lines, Yogev et al. [41] reported the MDC of SmO_2_ values of 16% to 18% at a high intensity, which are higher compared to our study. However, their data were obtained from the vastus lateralis muscle using a cycling protocol designed for trained cyclists, introducing methodological differences. Although few studies have examined the MDC of SmO_2_, available evidence suggests that data variability tends to be lower in smaller muscles, such as the gastrocnemius, due to their lower blood volume [43]. These findings highlight the need to interpret SmO_2_ values with caution due to their high variability, while emphasizing their potential for identifying training adaptations.

Also, this shows an inverse relationship between SmO_2_ and spirometric variables, which is logical, given that increased physical exertion requires more oxygen, potentially leading to a SmO_2_ decrease [44,45]. Additionally, IMU variables, such as acceleration, knee load, and especially angular velocity, show a negative correlation with SmO_2_. This finding aligns with Chalitsios et al. [18], who identified inverse relationships between SmO_2_ and variables measured by accelerometers placed on the lower tibia and metatarsals to analyze running mechanics. Our study is groundbreaking in correlating IMU sensor data with NIRS sensor data positioned laterally near the knee during treadmill running. This suggests that the SmO_2_ decrease is primarily influenced by speed rather than force vectors in various directions [46]. Also, the regression model indicates that angular velocities are inversely associated with SmO_2_ (β = −13.882, *p* < 0.001), with a significant inverse correlation prevailing in high-intensity zones (MIT: r = −0.376, *p* = 0.035) (see Figure 2). Conversely, knee load exhibits a positive relationship with SmO_2_, meaning that lower SmO_2_ values correspond to reduced force load on the knee. The SmO_2_ decrease depends on movement efficiency and intramuscular pressure rather than the magnitude of mechanical load derived from the accelerometer [47]. SmO_2_ cannot be a direct marker of knee loading, but it is a metabolic marker that must be complemented with external load measurements to accurately interpret performance [4]. This data analysis of SmO_2_ can be extrapolated to more intense movements and faster changes in direction. As indicated in other studies, SmO_2_ is more sensitive to changes in high-intensity running, such as the repeated sprint ability [4,10].

Finally, these findings highlight the SmO_2_ sensitivity for distinguishing training zones [2]. Moreover, both V’O_2_ and SmO_2_ are the most robust predictors of performance in incremental testing, confirming their fundamental role in aerobic capacity [45,48,49]. However, although they behave inversely, V’O_2_ remains the gold standard in spiroergometric testing, while SmO_2_ can complement it by identifying changes in peripheral metabolism. This is particularly relevant in high-intensity zones (>80% V’O_2_ max), where SmO_2_ continues to detect changes not captured by VO_2_ due to the cardiopulmonary oxygen plateau. These SmO_2_ changes are related to blood flow changes or the redistribution of blood flow [2,50].

### 4.1. Recommendations and Limitations

The combination of traditional physiological measures obtained in a laboratory setting (V’O_2_, V’E, and HR) with the use of NIRS and IMU sensors provides a more comprehensive understanding of physical effort. This integrated approach allows for the identification of factors limiting performance. However, the absence of field testing is a limitation of this study and would further interest researchers. Furthermore, the small sample size analyzed makes it difficult to extrapolate the results to all populations. Another point to note is that the inverse correlations of 0.45 and 0.37 may not be strong enough to guarantee predictive validity, as higher values (typically above 0.7) are generally considered strong associations. Similarly, the limited statistical power in some variables, particularly those related to SmO_2_ and IMU, may not be sufficient to rule out the possibility that certain effects occurred by chance, due to interindividual variability and the small sample size. Nevertheless, this study shows consistency in the observed trends and establishes initial relationships between key variables.

These results lay the groundwork for future studies with larger sample sizes and more specific populations, particularly in clinical and sports contexts, such as knee injury risk analysis. Moreover, a more robust dataset would enable the application of machine learning models, such as neural networks, Lasso, or ElasticNet, facilitating the development of integrated technologies. This could support the implementation of combined sensors (IMU, SmO_2_, and V’O_2_) within a single device, thereby optimizing real-time performance monitoring and injury prevention.

#### Practical Application

These findings offer a valuable tool for coaches and exercise physiologists by supporting the use of portable NIRS sensors to monitor muscle oxygenation in real time during efforts above 80% of V’O_2_max, where a 6% error margin is considered acceptable. Additionally, combining knee joint loading data with SmO_2_ provides a practical approach to monitoring internal and external load during explosive actions, such as sprints, accelerations, and decelerations. This enables more accurate, data-driven feedback for technical staff.

## 5. Conclusions

The results of this study indicate that V’O_2_ and SmO_2_ are the primary predictors of performance in a maximal incremental exercise test. Furthermore, a 6% error in SmO_2_ measurements can be considered acceptable for identifying physiological adaptations after exceeding 80% of V’O_2_max in a healthy population.

In addition, external loading variables of the knee joint measured with an IMU, and internal loading assessed via SmO_2_ NIRS sensors highlight an interaction that may be relevant for high-intensity activities, where angular velocity increases considerably, such as sprints, accelerations, and decelerations.

These findings open new lines of research into the potential use of SmO_2_ as a physiological marker, particularly in relation to the energy demand of the muscles involved in knee movement.

## Figures and Tables

**Table 1:** Description of spiroergometric parameters, IMU, and SmO_2_ during a maximal incremental exercise test.

		LIT	LIT	LIT	MIT	HIT	HIT	SmO_2_ Difference Between Training Zones
Variables	Resting	50–59%	60–69%	70–79%	80–89%	90–99%	100%	
Time (sec)	0 ± 0	77 ± 40	141 ± 79	204 ± 109	299 ± 132	367 ± 157	447 ± 187	MIT vs. LIT (80–89% ≠ 50% and 69%). HIT vs. LIT (90–100% ≠ 50–79%) HIT vs. MIT (No difference)
V’O_2_ mL/min	857 ± 277	2119 ± 461 *	2581 ± 587	2958 ± 733	3315 ± 728	3619 ± 892	4089 ± 844	
V’O_2_ mL/kg/min	13.9 ± 4.1	33.2 ± 4.3 *	40.3 ± 6.2	45.9 ± 6.7	51.6 ± 7.6	56.2 ± 9.7	63.7 ± 9.5	
V’E (L/min)	31.4 ± 8.6	59.7 ± 12.9 *	66.7 ± 13.7	80.1 ± 19.0	98.4 ± 22.7	108.9 ± 23.9	124.6 ± 23.7	
HR (bmp)	117 ± 20	151 ± 19 *	163 ± 15	171 ± 13	179 ± 11	184 ± 10	190 ± 8	
ACC (m/s^2^)	2.29 ± 0.77	2.58 ± 0.68	2.99 ± 0.87	3.33 ± 0.64	3.47 ± 0.68	3.61 ± 0.81	4.09 ± 0.79	
Knee Load (U.A)	255 ± 89	314 ± 106	334 ± 86	340 ± 85	371 ± 92	419 ± 104	447 ± 108	
Angular Velocity (s/°)	24.2 ± 6.3	27.0 ± 5.8	30.0 ± 7.4	34.2 ± 5.9	36.6 ± 5.2	40.8 ± 5.6	42.3 ± 6.4	
SmO_2_ (%)	61.1 ± 14.8	48.1 ± 12.5 *	44.0 ± 14.7	31.8 ± 9.1	21.5 ± 9.2	14.3 ± 10.2	13.2 ± 10.9	

Note. LIT = low-intensity training; MIT = moderate-intensity training; HIT = high-intensity training. Difference between percentages with the previous stage (*). V’O_2_ = oxygen consumption, V’E = ventilation, HR = heart rate, ACC = horizontal acceleration, and SmO_2_ = muscle oxygen saturation.

**Table 2:** Reliability and sensitivity of muscle oxygen saturation during a maximal incremental exercise test.

Variables	SmO_2_		Reliability		Sensitivity	
	Test 1	Test 2	ICC	CV%	SE	MDC
Resting	57.9 ± 15.1	64.0 ± 11.5	0.143	21.8	3.5	9.7
50%	48.2 ± 11.7	50.1 ± 11.6	0.744	23.7	3.1	8.6
60%	44.6 ± 14.2	46.6 ± 12.5	0.527	29.2	3.5	9.7
70%	30.6 ± 9.2	31.6 ± 8.8	0.568	28.9	2.4	6.7
80%	18.3 ± 6.9	18.8 ± 5.6	0.577	33.6	1.6	4.4
90%	8.9 ± 5.1	9.8 ± 4.3	0.594	50.2	1.2	3.3
100%	9.1 ± 7.4	10.2 ± 7.6	0.729	77.7	2.0	5.5

Note. ICC = intraclass correlation coefficient, CV% = coefficient of variation, SE = standard error, and MDC = minimum detectable change.

**Table 3:** Correlation of spiroergometric parameters and IMU with muscle oxygen saturation during a maximal incremental exercise test.

Variables	SmO_2_ (%)	VO_2_ [mL/kg/min]	VE [L/min]	HR (bmp)	Acc (m/s^2^)	Player Load (U.A.)	Angular Velocity (S/°)
SmO_2_ (%)	—						
V’O_2_ [mL/kg/min]	−0.799 *	—					
V’E [L/min]	−0.800 *	0.899 *	—				
HR (bmp)	−0.783 *	0.814 *	0.794 *	—			
Acc (m/s^2^)	−0.455 *	0.479 *	0.420 *	0.269 *	—		
Knee Load (U.A.)	−0.379 *	0.344 *	0.345 *	0.241 *	0.801 *	—	
Angular Velocity (S/°)	−0.617 *	0.651 *	0.713 *	0.467 *	0.694 *	0.613 *	—

Note. *p* value < 0.05 * statistically significant. Pearson correlation interpretation: 0.0–0.1 = trivial, 0.1–0.3 = small, 0.3–0.5 = moderate, 0.5–0.7 = large, 0.7–0.9 = very large, and 0.9–1 = almost perfect. V’O_2_ = oxygen consumption, V’E = ventilation, HR = heart rate, ACC = horizontal acceleration, and SmO_2_ = muscle oxygen saturation.

**Table 4:** Linear regression model to estimate performance during a spiroergometric test using IMU and NIRS sensors.

Model Coefficients—Time (seg)				
Predictor	Estimate (β)	SE	t	*p*
Intercept	217.3	133.8	1.62	0.107
V’O_2_ [mL/kg/min]	10.2	1.3	7.65	<0.001 *
V’E [L/min]	−0.632	0.624	−1.01	0.313
HR (bmp)	−1.1	0.669	−1.65	0.100
Acc (m/s^2^)	−30.7	17.2	−1.79	0.076
Knee Load (U.A.)	−0.248	0.127	−1.96	0.053
Angular Velocity (S/°)	2.8	1.7	1.68	0.095
SmO_2_ (%)	−3.7	0.714	−5.28	<0.001 *

Note. *p* value < 0.05 * statistically significant and SE = standard error and β = beta coefficient. V’O_2_ = oxygen consumption, V’E = ventilation, HR = heart rate, ACC = horizontal acceleration, and SmO_2_ = muscle oxygen saturation.

## Data Availability

The data of this study are available upon request from the corresponding author.
